# Detecting internally symmetric protein structures

**DOI:** 10.1186/1471-2105-11-303

**Published:** 2010-06-03

**Authors:** Changhoon Kim, Jodi Basner, Byungkook Lee

**Affiliations:** 1 Laboratory of Molecular Biology, Center for Cancer Research, National Cancer Institute, National Institutes of Health, Bldg. 37, Room 5120, 37 Convent Dr MSC 4264, Bethesda MD 20892-4264 USA; 2 Bioinformatics & Molecular Design Research Center, Yonsei Engineering Research Park, Yonsei University, 262 Seongsanno, Seodaemun-gu, Seoul 120-749, Korea

## Abstract

**Background:**

Many functional proteins have a symmetric structure. Most of these are multimeric complexes, which are made of non-symmetric monomers arranged in a symmetric manner. However, there are also a large number of proteins that have a symmetric structure in the monomeric state. These internally symmetric proteins are interesting objects from the point of view of their folding, function, and evolution. Most algorithms that detect the internally symmetric proteins depend on finding repeating units of similar structure and do not use the symmetry information.

**Results:**

We describe a new method, called SymD, for detecting symmetric protein structures. The SymD procedure works by comparing the structure to its own copy after the copy is circularly permuted by all possible number of residues. The procedure is relatively insensitive to symmetry-breaking insertions and deletions and amplifies positive signals from symmetry. It finds 70% to 80% of the TIM barrel fold domains in the ASTRAL 40 domain database and 100% of the beta-propellers as symmetric. More globally, 10% to 15% of the proteins in the ASTRAL 40 domain database may be considered symmetric according to this procedure depending on the precise cutoff value used to measure the degree of perfection of the symmetry. Symmetrical proteins occur in all structural classes and can have a closed, circular structure, a cylindrical barrel-like structure, or an open, helical structure.

**Conclusions:**

SymD is a sensitive procedure for detecting internally symmetric protein structures. Using this procedure, we estimate that 10% to 15% of the known protein domains may be considered symmetric. We also report an initial, overall view of the types of symmetries and symmetric folds that occur in the protein domain structure universe.

## Background

Many protein chains are made of repeating units of similar structure, which are often arranged in a beautifully symmetric manner. Some well-known examples are the 8-fold symmetric "TIM" barrel folds, the β-blade propellers, the α/α superhelices, the leucine-rich repeat horseshoe-shape structures, etc. (See, for example, a review by Andrade et al. [1])

Occurrence of symmetric structures poses a number of questions: What sequence and energetic features make repeating units fold into a similar structure and cause them to arrange in a symmetric pattern? What is the biological function of such symmetric chains? How are they different from the symmetric structures of multimeric complexes, which are formed by symmetrically assembling non-symmetric monomers? How many symmetric chains and what types of symmetry exist in the protein universe? What is their evolutionary history?

Symmetric structures also tend to cause problems for automatic domain partition programs, which may recognize a single repeating unit or several such units as a domain for some chains and the whole repeat set with the full symmetry for others of the similar structure. For structures with super-helical symmetry, automatic structure comparison can be a problem also because of the flexibility of the structure between the repeating units. One or a few units in two such structures can be recognized as similar, but the whole structures can often be sufficiently different for routine detection of the similarity.

In order to answer some of the questions posed above, we need to collect as many symmetric protein samples as possible. It is also useful to identify symmetric structures and separate them from the non-symmetric structures before an automatic structure comparison or domain partition operation. For these and other reasons, a number of procedures have been developed for identifying symmetric structures over the years. These procedures can be broadly classified into two groups.

One class of methods finds repeats by using a structure alignment program that can report not just one optimal alignment but many other independent, sub-optimal alignments between a pair of proteins. Repeats are found by running such a program on a protein and its own copy to find non-trivial self-alignments. An early work using this principle is by Kinoshita et al. [2] but more recent works by others [3-6] use essentially the same principle. These methods depend on the ability of the structure comparison programs to find sub-optimal, yet still significant, structural alignments. Each individual repeat is found independent of others and the fact that the same motif is repeated in a regular pattern is not explicitly used.

Another class of methods explicitly exploits the periodic occurrence of repeats along the primary sequence. The method by Taylor et al. [7], its sequel DAVROS [8] and the method by Murray et al. [9] all start from the *N*-by-*N *SAP [10] matrix, where *N *is the number of residues in the protein. Each element of this matrix gives a measure of the similarity of the structural environments of a pair of residues. Two segments of similar structures, without a gap in the sequence, will appear as two symmetrical diagonal lines of high scores in this matrix. The methods detect periodic occurrence of such lines using different mathematical devices (Fourier and wavelet transforms). The method by Chen et al. [11] is partly similar in that it also makes use of the periodic features of an N-by-N matrix, although their matrix is different. Each matrix element in this case represents a sub-sequence of given length and position in the primary sequence. The value of the matrix element is one if the structure of the corresponding sub-sequence is found to be similar to that of at least one other sub-sequence of the same length, thus requiring an un-gapped structural similarity, and zero otherwise. Since the prominent features appear differently in this matrix than in the SAP matrix above, a couple of different methods are used to recognize the repeats. At least one of these (the Pearson correlation) again depends on the periodic occurrence of prominent features along the primary sequence. Generally, this class of methods may depend too much on the regularity of the repeats; insertions or deletions, either within or between the repeats, reduce the signal and will make the detection difficult.

Here we describe a different method, SymD for Symmetry Detection, which makes use of the symmetry of the structure. In this method a protein structure is aligned to itself after circularly permuting the second copy by all possible number of residues. For each circular shift, we keep only one optimal, non-self structural alignment, fully allowing gaps and unaligned loops. We call this process the alignment scan. This has the effect of amplifying the signal for the symmetric transformation of the structure. Suppose the structure is two-fold symmetric. Call the two similar parts A and B. All known procedures, with a possible exception of the methods that use OPAAS [6,12], will report the similarity of A to B and B to A separately, with a score corresponding to matching *N/2 *residues. However, if the second copy is circularly permuted by *N/2 *residues, it has the structure B-A, and since the structure is symmetric, it will match the original structure A-B in its entirety. Therefore, the alignment scan will report a score corresponding to *N *matched residues at the position *N/2 *against a background of no significant alignment at all other shift values. From the superposition matrix at the position of the maximum score, we also get the rotation angle (180° in this case), the position and orientation of the symmetry axis, and the translation along the symmetry axis when the symmetry is that of a helix. The boundaries of repeating units, and in fact all the residues that make up each repeating unit, can be obtained from the structure-based sequence alignment between the original and the circularly permuted sequences. We report some individual sample cases and also some statistics on the occurrence of symmetric structures found in the SCOP1.73 ASTRAL 40% set [13] using this procedure.

## Results

### Z-scores from alignment scans

For a protein with *N *residues, the SymD procedure performs *N-3 *structure alignments (alignment scan), each of which starts from the initial alignment which forces the original sequence to align with the same sequence but circularly permuted by *n *residues where *n *ranges from 1 to *N-3*. This initial alignment is refined by structure superposition and sequence alignment cycles. (See Methods.)

**Figure 1:**
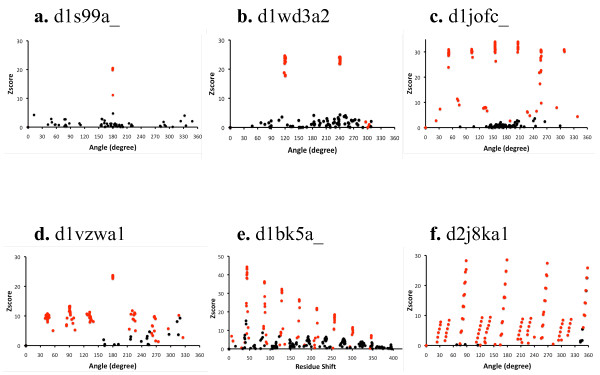
**Z-scores from alignment scans of 6 sample domains**. The Z-score vs. rotation angle scatter plot for all alignments from the alignment scan for (a) d1s99a_, (b) d1wd3a2, (c) d1jofc_, (d) d1vzwa1, and (f) d2j8ka1. The red points are those whose rotation axis is within about 20° (cosθ > 0.95) of that of the point with the highest Z-score. Others are in black. Panel (e) for d1bk5a_, is an exception; here the Z-scores are plotted against the average alignment shift (average of the residue serial number differences between aligned residues). Note that points with negative Z-scores are not shown. Note also that the self-alignment peak, which would occur at angle 0° or zero average shift, is not visible in these plots. This is because the aligned pairs between residues whose serial numbers differ by 3 or fewer residues were not included in the T-score calculation (see Methods).

**Figure 2:**
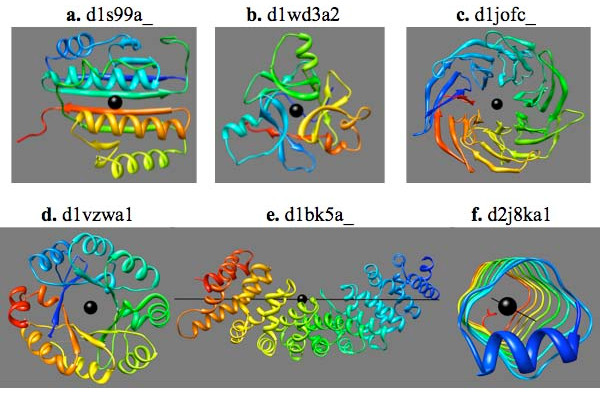
**Structures of the 6 sample domains**. The ribbon rendering of the structure of the proteins of Fig. 1: (a) d1s99a_, a 2-fold symmetric ferredoxin-like fold, (b) d1wd3a2, a 3-fold symmetric beta-trefoil, (c) d1jofc_, a 7-bladed beta-propeller, (d) d1vzwa1, a 2-fold symmetric TIM barrel, (e) d1bk5a_, an alpha/alpha superhelix, and (f) d2j8ka1, a right-handed beta-helix with square cross-section. The ribbons are colored in rainbow colors, starting from blue for the N-terminus and ending with red at the C-terminus. The calculated symmetry axis is shown as a black rod with a ball at the center in this and in all other figures.

Fig. 1 shows the Z-scores based on the T-score (a weighted number of aligned residues, see Methods) of all refined alignments from an alignment scan for six sample structures. The structures are shown in Fig. 2.

D1s99a_ is the product of the *B. subtilis *YkoF gene, which is involved in the hydroxymethyl pyrimidine (HMP) salvage pathway. The structure (Fig. 2, panel a) is made of 2-fold symmetric, tandem repeats of a ferredoxin-like fold [14]. Panel (a) of Fig. 1 is a scatter plot of the Z-score vs. the rotation angle for all the alignments from the alignment scan. It clearly shows one angle, 180°, at which the Z-scores are much larger than at any other angles.

D1wd3a2 is the arabinose-binding domain of alpha-L-arabinofuranosidase B from *Aspergillus kawachii*. It has the structure (Fig. 2, panel b) of the 3-fold symmetric beta-trefoil fold [15]. The Z-score versus rotation angle scatter plot (Fig. 1, panel b) shows two peaks, at 120° and 240°, respectively.

D1jofc_ is a muconate lactonizing enzyme from *Neurospora crassa*. It has the 7-fold symmetric 7-bladed beta-propeller structure (Fig. 2, panel c), in which the C-terminal end of the molecule comes near the N-terminus to complete the first repeating unit [16]. Therefore, when the sequence of the duplicated structure is circularly permuted by one repeating unit, all units align nearly as well as when the second sequence is not shifted at all. The resulting Z-score plot (Fig. 1, panel c) shows 6 peaks of nearly the same height at angles that are nearly multiples of 360°/7 = 52°.

D1vzwa1 is a bispecific phosphoribosyl isomerase (PriA) from *Streptomyces coelicolor*. It has a (β/α)_8 _TIM barrel structure (Fig. 2, panel d) with a 2-fold symmetry [17] and the less perfect 8-fold symmetry of the (β/α)_8 _barrels. The Z-score plot (Fig. 1, panel d) reflects these symmetries: It shows one major peak at 180° and a lower, but clearly recognizable, set of peaks at 45° intervals.

D1bk5a_ is the major portion of yeast Karyopherin alpha, which is a selective nuclear import factor. It has an ARM-type alpha-alpha superhelical repeat structure (Fig. 2, panel e), in which 10 repeating units of 3 alpha-helices each (except for the first unit which has only 2 helices) are arranged in a superhelical manner [18]. The Z-score plot shows 8 successively decreasing peaks at about 43 residue intervals (Fig. 1, panel e). A superhelical structure is an open structure in which the N- and C-terminals are at opposite ends of the molecule. For such a structure, shifting the sequence of one molecule by a repeating unit reduces the number of matching units by one even when the sequence is circularly permuted. This explains the decrease in peak height at successively larger shifts. Ideally, there would be 9 peaks since the structure contains 10 repeats. However, the last peak is weak and does not rise above the background level.

Notice that the Z-scores are plotted against the average alignment shift rather than the rotation angle for this structure. The average alignment shift [19] is the average of the residue serial number differences between aligned residue pairs after the alignment has been fully refined by RSE. For this structure, the Z-score plot shows a more regular pattern when plotted against the average alignment shift than when plotted against the rotation angle (plot not shown). This implies that the number of residues is more conserved than the relative orientation between the repeats in this structure.

D2j8ka1 is a protein made by the fusion of two pentapeptide repeat proteins, Np275 and Np276, from *Nostoc Punctiforme *[20]. It has the structure of a parallel beta-helix with a square cross-section (Fig. 2, panel f). Let us label the successive corners of the square cross-section as C_1_, C_2_, etc. The basic symmetry of the structure, call it H_1_, is a helical operation that matches C_n _to C_n+1_. The rotation angle of this symmetry is about 90° and the translation is small (1.3 Å, see below). The structure also has the symmetry H_2 _= H_1_^2^, which matches C_n _to C_n+2_, H_3 _= H_1_^3^, which matches C_n _to C_n+3_, etc. Among these compounded symmetries, the 4-repeat symmetry, H_4 _= H_1_^4^, is special since this operation matches one layer to the next layer. This is nearly a pure translation (of about 4.4 Å, see below), which essentially corresponds to the separation between the two parallel beta-strands. It has a small rotation component of -2° (see below), which represents the slight left-handed twist between successive layers (Fig. 2, panel f).

The Z-score plot that SymD program gives for this structure is shown in Fig. 1, panel f. Concentrating on only those points for which the Z-score is >10, the plot shows 4 sets of points, each set at approximately 90°, 180°, 270° or 360°. These sets correspond to the square cross-section of the structure. The point with the highest Z-score in the first set has the rotation angle of 88.6° and the translation of 1.29 Å. This is the H_1 _symmetry. The other points in the same set represent H_1_*H_4_, H_1_*H_4_^2^, etc. Similarly, the set of points in the second set, at around 180°, represents symmetries H_2_, H_2_*H_4_, H_2_*H_4_^2^, etc. and the third set at around 270° represents symmetries H_3_, H_3_*H_4_, H_3_*H_4_^2^, etc. The point with the highest Z-score in the last set of points has the rotation angle of -1.9° and the translation of 4.42 Å. This represents the H_4 _symmetry, which essentially translates the whole molecule along the helix axis by one layer, with a small left-handed twist. The other points in the same set at successively larger negative angles represent H_4_^2^, H_4_^3^, etc.

### Number of symmetric structures in the universe of protein structural domains

The symmetries detected here are all pseudo or approximate symmetries. The imperfections in the symmetry arise both because the repeating units are not all exactly the same and because they are not arranged in a perfectly symmetrical manner. Therefore, the notion of symmetric structure requires using a cutoff value of some variable. We used the Z-score (see Methods) for this purpose.

**Figure 3:**
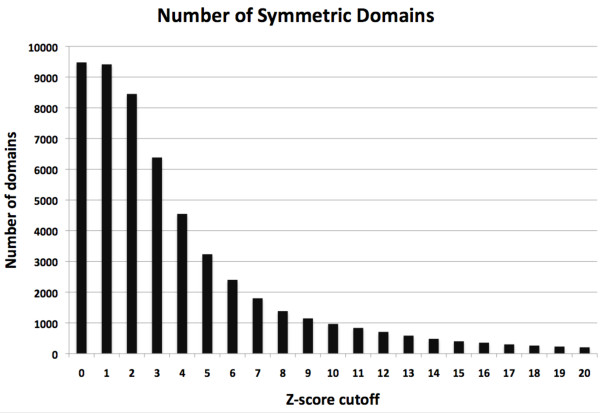
**Number of symmetric domains vs. Z-score cutoff value**. The height of the bars give the number of proteins in the ASTRAL 40 domain dataset that have a Z-score higher than the cutoff value given by the X-axis.

SymD was run on all 9479 domains in the SCOP1.73 ASTRAL 40% set downloaded from the ASTRAL website http://astral.berkeley.edu/. The results of these runs (the highest Z-score and the rotation angle and the translation for the alignment with the highest Z-score) for all domains are given in the additional file 1: all_domains.xls. Fig. 3 shows the number of proteins that have a Z-score above the value indicated along the X-axis. At a sufficiently low Z-score cutoff value, nearly all proteins have a Z-score higher than the cutoff value. As the Z-score cutoff value is raised, the number of proteins with a Z-score higher than the cutoff value initially decreases rapidly and then more gradually at higher cutoff values. The number changes smoothly, indicating that there are proteins with all degrees of perfection of symmetry.

**Figure 4:**
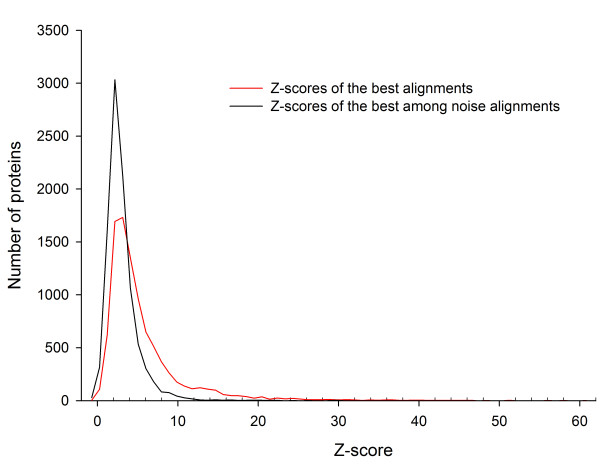
**Z-score distributions**. Distribution of the Z-scores of the 'best' alignments (red) and the best among the 'noise' alignments (black). The number of 'noise' alignments is small above Z-score of 10 and rises sharply below Z-score of 8.

A better feel for the relation between symmetry and the Z-score can be obtained by considering the Z-score distribution of the alignments that are not likely to represent the symmetry of the protein. We selected two alignments among the *N-3 *refined alignments from the alignment scan for each protein: the 'best' alignment and the best among the 'noise' alignments. The 'best' alignment is the one with the highest Z-score. The 'noise' alignments are those whose rotation axis makes more than about 20° angle (cosine of the angle less than 0.95) to that of the 'best' alignment. Fig. 4 shows the Z-score distribution among the 9479 proteins for the 'best' (red) and for the best 'noise' (black) alignments. The black curve shows that there are few 'noise' alignments with the Z-score above 10 and that the number increases sharply when Z-score falls below 8.

On this basis, we consider a protein to be symmetric when the Z-score of its 'best' alignment is above 8 or 10. The number of symmetric domains in the ASTRAL40 dataset is then 968 (10%) or 1385 (15%) depending on whether the Z-score cutoff value used is 10 or 8, respectively.

### Symmetric SCOP folds

There are 1081 Folds in the ASTRAL 40% dataset we used. The number of symmetric domains in each of these Folds, using the Z-score cutoff values of 8 and 10, are given in the additional file 2: all_folds.xls. Many SCOP Folds contain both the domains that are judged to be symmetric and those that are not. The number of SCOP folds that contain at least one, 50%, or 100% of domains that are symmetric, using the z-score cutoff criteria of 10 or 8, are given in Table 1. For example, 8% or 13% of all SCOP Folds contain half or more domains that are judged to be symmetric. But many of these are singletons and other small folds (folds with small number of domains). If one considers only the 198 Folds that contain at least 10 domains, the number of Folds of which half or more of the domains are judged to be symmetric is 21 (11%) or 31 (16%) depending on the Z-score cutoff value used.

**Table 1:** Number of SCOP Folds that contain symmetric domains

	Among all 1081 folds		Among 198 folds with more than 10 domains	
	
	Z > 10	Z > 8	Z > 10	Z > 8
At least 1^a^	156 (14%)	230 (21%)	66 (33%)	94 (47%)

50% or more^b^	90 (8%)	136 (13%)	21 (11%)	31 (16%)

All^c^	45 (4%)	74 (7%)	3 (2%)	6 (3%)

^a ^Number of folds that contain at least one domain with the indicated z-scores.

^b ^Number of folds of which 50% or more domains have the indicated z-scores.

^c ^Number of folds of which all domains have the indicated z-scores.

**Figure 5:**
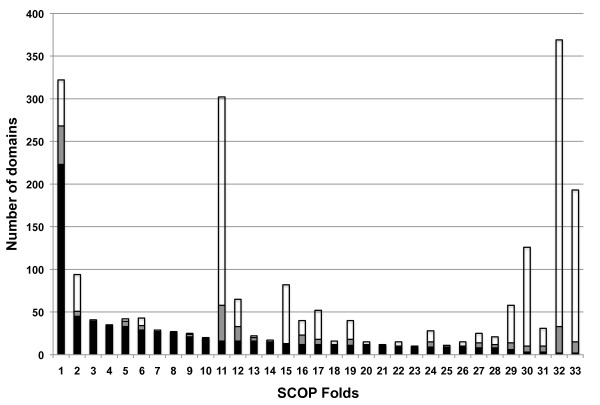
**SCOP Folds that contain at least 10 symmetric domains**. There are 33 SCOP Folds that contain at least 10 domains with a Z-score of 8 or better. The X-axis represents these Folds. For each of these Folds, the Y-axis gives the total number of domains in the Fold (tip of the white bar), the number of domains with Z-score of 8 or better (tip of the grey bar), and the number of domains with Z-score of 10 or better (tip of the black bar). The Folds along the X-axis were sorted according to the height of the black bars.

There are 23 or 33 Folds that contain 10 or more symmetric domains, using the Z-score cutoff value of 10 or 8, respectively. Fig. 5 shows the total number of domains and the number of symmetric domains in the 33 folds. The names of these folds are listed in Table 2.

**Table 2:** Names of SCOP Folds shown in Fig. 5

**SN**^**a**^		Scop Id	**Zscore** ** >= 8**^**b**^	**Zscore** **>= 10**^**c**^	**Total**^**d**^	Fold Name
1		c.1	268	223	322	TIM beta/alpha-barrel
2		a.118	51	45	94	alpha-alpha superhelix
3		b.42	41	39	41	beta-Trefoil
4		b.69	35	35	35	7-bladed beta-propeller
5		a.102	39	33	42	alpha/alpha toroid
6		a.25	34	29	43	Ferritin-like
7		b.80	27	27	29	Single-stranded right-handed beta-helix
8		b.68	27	27	27	6-bladed beta-propeller
9		c.10	24	21	25	Leucine-rich repeat, LRR (right-handed beta-alpha superhelix)
10		f.4	19	18	20	Transmembrane beta-barrels
11		d.58	58	16	302	Ferredoxin-like
12		a.24	33	16	65	Four-helical up-and-down bundle
13		d.131	20	16	22	DNA clamp
14		d.211	15	14	17	beta-hairpin-alpha-hairpin repeat
15		b.82	13	13	82	Double-stranded beta-helix
16		c.94	18	12	52	Periplasmic binding protein-like II
17		a.2	18	12	40	Long alpha-hairpin
18		b.81	12	12	16	Single-stranded left-handed beta-helix
19		a.7	23	11	40	Spectrin repeat-like
20		c.93	12	11	15	Periplasmic binding protein-like I
21		d.126	12	11	12	Pentein, beta/alpha-propeller
22		d.19	10	10	15	MHC antigen-recognition domain
23		b.67	10	10	10	5-bladed beta-propeller
24		a.26	15	9	28	4-helical cytokines
25		a.47	11	9	11	STAT-like
26		b.50	10	9	15	Acid proteases
27		a.29	14	8	25	Bromodomain-like
28		d.157	12	8	21	Metallo-hydrolase/oxidoreductase
29		a.39	14	6	58	EF Hand-like
30		b.40	10	3	126	OB-fold
31		d.32	10	3	31	Glyoxalase/Bleomycin resistance protein/Dihydroxybiphenyl dioxygenase
32		b.1	33	2	369	Immunoglobulin-like beta-sandwich
33		c.2	15	2	193	NAD(P)-binding Rossmann-fold domains

^a^Serial number of folds in Fig. 5.

^b^Number of domains with Z-score >= 8.

^c^Number of domains with Z-score >= 10.

^d^Total number of domains in the fold.

**Figure 6:**
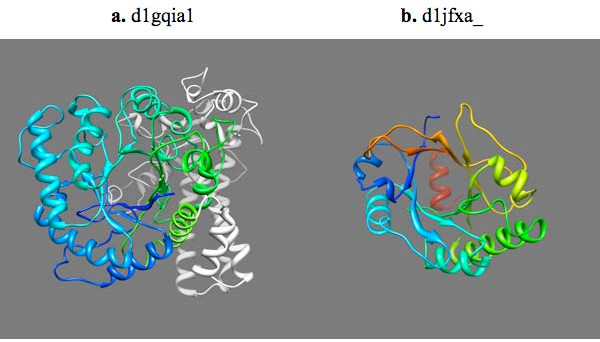
**Two TIM barrel domains with low Z-scores**. (a) D1gqia1 with Z-score of 3.03, which is the lowest among all Z-score of TIM barrel domains in the ASTRAL40 dataset. The domain has 561 residues, of which only the N-terminal 320 residues, colored in different shades of blue to green, may be considered to constitute the symmetric core. The remaining 241-residue C-terminal extension, colored white, is not symmetric. (b) D1jfxa_ with the Z-score of 7.20, which is near the lower cutoff value of 8, in rainbow color from blue for the N-terminus to red for the C-terminus. This structure has a distorted TIM barrel fold: the fifth helix (yellow) is extended and barely discernible, 6^th^, 7^th^, and 8^th ^helices are missing and the 8^th ^strand is in reverse direction, being anti-parallel to its neighboring 7^th ^and 1^st ^strands.

The fold with the most symmetric domains is the (β/α)_8 _TIM barrel fold. SymD finds 223 (69%) to 268 (83%) of the 322 domains of this fold symmetric depending on the Z-score cutoff value used. Many of the structures with low Z-score are non-symmetric because of the presence of one or more large extensions outside of the barrel. Others have distorted barrels. Two examples of such structures are shown in Fig. 6, to be compared with a symmetric TIM barrel shown in Fig. 2d.

The alpha-alpha superhelix fold (Fig. 2e) has the second most number of symmetric domains. Many of the domains in this fold that have low Z-scores are either short or contain collections of helical hairpins that do not maintain a superhelical symmetry.

All 41 beta-trefoil domains (Fig. 2b) are found to be symmetric at the Z-score cutoff level of 8 and all but 2 at the level of 10. All the beta-propeller domains (Fig. 2c) are found to be symmetric at the Z-score cutoff level of 10. The 4- to 7-blade propellers are included in Fig. 5; the six 8-bladed beta-propellers are not shown. All but two of the 20 transmembrane beta-barrels are recognized as symmetric at the Z-score cutoff level of 10 and all but one at the level of 8. It should be noted that the beta-hairpins in these structures are often not all of the same lengths and some strands in some structures make excursions into the inside of the barrel.

SymD finds only a relatively small fraction of domains with the Ferredoxin fold symmetric. The basic unit of the Ferredoxin fold is an alpha-helix followed by a beta-hairpin. A typical Ferredoxin fold contains two of these units, which ideally are related by a two-fold symmetry. However, many structures are circularly permuted and have an extension at the N- and/or C-terminus of the molecule. Also, the two helices are often positioned and oriented in an asymmetric manner. The most symmetric ones in this fold actually have four basic units, of which the symmetry relates one pair to the other pair. (See, for example, Fig. 2a.)

**Figure 7:**
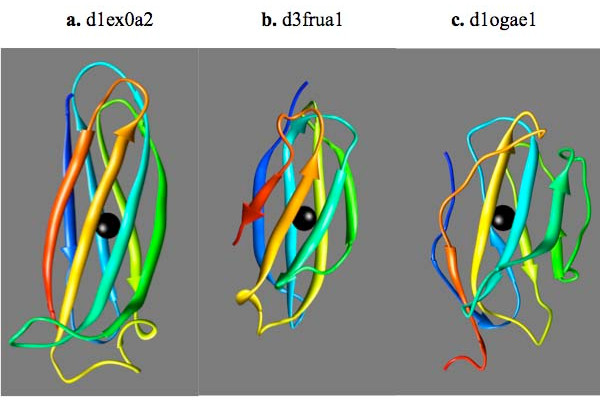
**Three domains with the immunoglobulin fold with high and medium Z-scores**. (a) D1ex0a2 (Z-score = 10.9, the highest). (b) D3frua1 (Z-score = 10.4, the second highest). (c) D1ogae1 (Z-score = 5.753, near the median). Each structure is colored in rainbow colors from blue for the N-terminus to red for the C-terminus. The viewing direction is down the calculated symmetry axis, indicated by black rod, which is not visible because of the black ball placed at the center of the axis.

The fold with the largest number of domains in the ASTRAL domain dataset is the immunoglobulin-like beta-sandwich fold (fold #32 in Fig. 5). SymD finds most of the domains in this fold as non-symmetric. Only two out of 369 domains have the Z-score greater than 10. These are shown in Fig. 7 along with another protein with a median Z-score for comparison.

## Discussion

### Characteristics of the SymD procedure

The simple method of doing the alignment scan using permuted structure has at least a couple of advantages compared to other reported methods. One is that at each stage, it finds the best alignment allowing gaps and unaligned regions. This is a significant advantage because repeating units in a symmetric structure often contain loops of varying lengths. Other algorithms that use information on the regularity of repeats [7-9,11] assume absence of long gaps. Obviously, the length of the insertion matters: If the insertion is sufficiently large, the whole structure becomes non-symmetric because the non-symmetric inserted part can no longer be ignored. In such cases, SymD will also declare the structure non-symmetric, not because it does not find the symmetric repeating units but because the symmetric part is too small a part of the whole structure.

Another advantage is that this algorithm enhances positive signal by making use of the symmetry. For example, suppose a structure has six repeating units arranged in a 6-fold symmetric manner, but one unit is rather different from the rest. Most programs will fail to recognize the 6-fold symmetry because this one unit does not align well with the others. In contrast, SymD will find that 4 of the 6 units match at every 6-fold position and could very well report the protein as symmetric. (Here again, the degree to which the one unit differs from the rest matters. If the one unit is not too different and the protein may still be considered symmetric overall, SymD will correctly declare the protein symmetric. On the other hand, if the one unit is very different, the whole structure is not truly 6-fold symmetric and yet SymD could still declare it symmetric on the strength of the 4 matches out of 6. But we do not expect this to happen very often because, if one unit is so very different, it is likely that the rest of the molecule would also deviate from the 6-fold symmetry and the SymD Z-score would correspondingly deteriorate.)

These two features probably contribute to the remarkable fact that SymD finds, for example, all 83 beta-propeller structures in the ASTRAL 40% dataset as symmetrical without exception (see Results).

On the other hand, SymD algorithm is designed to detect global symmetry of a protein structure. If the protein contains a symmetric part, but is not symmetric as a whole because of the presence of other parts or other domains, SymD will tend to declare the protein non-symmetric and not recognize the symmetric sub-structure. The algorithm can probably be modified to recognize local symmetry, but this will be the subject of a future study.

### Number of symmetric proteins in the ASTRAL40 domain dataset

The number of symmetric proteins in a database depends both on the property one uses to measure the degree of symmetry and on the cutoff value of this property. We used in this study the Z-score of the T-score, which is a weighted number of aligned residues. In order to convert the T-score to the Z-score, we used a background distribution that was obtained from alignments of similar sized proteins whose rotation axis is significantly different from that of the best alignment (see Methods). A better method would probably use information other than just the size of the protein. For example, one could use protein class or secondary structure content information or perhaps each protein-specific background. We have not explored other quantities or other ways of obtaining the Z-score in this study.

Using the Z-scores, we found that 10% or 15% of the proteins in the ASTRAL40 dataset are symmetric depending on whether the Z-score cutoff value of 10 or 8 is used, respectively. This is comparable to the 14% found for proteins that contain duplicated sequence segments in all known protein sequences [21].

The number of symmetric folds is even more difficult to decide because many folds contain both symmetric and non-symmetric domains. But the fraction of symmetric folds is roughly in the similar range using a couple of different measures. For example, the fraction of folds that contain more than 50% of symmetric domains is 11% or 16%, using the two Z-score cutoff values, among the 198 folds that contain 10 or more domains (Table 1).

### Comparison with other programs

The number of symmetric proteins found by SymD is compared with that of several other programs in Table 3. The comparison is not completely satisfactory because the datasets used are different and only small number of numerical results are available in most cases. However, the Table shows that SymD finds a similar or more number of domains as symmetric, except when compared to GANGSTA+ by Guerler et al. [5], which finds more ferredoxin-like and immunoglogulin-like domains as symmetric than SymD does.

**Table 3:** Comparison with other methods

					# or % symmetric proteins found	
Program name		Dataset		Fold name		
					Others	SymD (SCOP 1.73, ASTRAL 40)
DAVROS [9]		CATH		LRR	89%	**84-96%**
				TIM barrels	60%	**69-83%**
				β-trefoils	67%	**95-100%**

OPAAS [6]		SCOP 1.55		β-propellers	86%	**100%**
				β-trefoils	72%	**95-100%**

Swelfe [4]		ASTRAL 50			172^¶^	**968-1385**

RQA [11]		SCOP 1.69		β-propellers	75%	**100%**
				β-trefoils	86%	**95-100%**

GANGSTA+[5]		SCOP 1.73,		ferredoxin-like	68	**16-58**
		ASTRAL 40		immunoglobulin-like	31	**2-33**
				β-trefoil	23	**39-41**
				four helical up-and-down bundle	16	**16-33**
				DNA clamp	14	**16-20**
				7-bladed b-propellers	13	**35**
				TIM barrels	12	**223-268**
				γ-crystalline-like	10	**7-9**

¶ Repeat length >50.

Since Guerler et al. used the same dataset as we did and the full results are available on their web site, we made a more detailed comparison for the 8 folds listed in their Table 1. The results of this comparison are given in the additional file 3: SymDGangstaData.xls, which gives the numerical values of the symmetry measures (Z-score for SymD and the fraction of sequentially aligned residues for GANGSTA+) by both programs for each domain of each fold, additional file 4: SymDGangsta.ppt, which gives the scatter plots of the symmetry measures, and additional file 5: SymDGangstaTable.xls, which gives a summary table of the numbers of domains that are considered symmetric or non-symmetric by the two programs. The scatter plots show generally good correlation between the two symmetry measures except for the four-helix up and down bundle fold. (See SymDGangsta.ppt.) The number of symmetric proteins depends on the cutoff value used for both methods. For the ferredoxin-like and the immunoglobulin-like folds, only a small adjustments in the cutoff values will be sufficient to make the number of symmetric proteins the same. (See SymDGangsta.ppt file.) However, the correlation is not perfect and the list of symmetric proteins would not be the same even after the cutoff values were adjusted to make the number of symmetric proteins the same. For the beta-trefoil, 7-bladed beta-propeller, and the TIM barrel folds, small adjustments of the cutoff values will not bring the number of symmetric proteins the same.

### Types of symmetry

We observed many different types of symmetry by visual inspection of the structures. The pattern of the Z-scores given by alignment scan procedure contains the symmetry information as described in the Results section. We are currently working on developing a robust, automatic procedure for determining symmetry types from such data.

**Figure 8:**
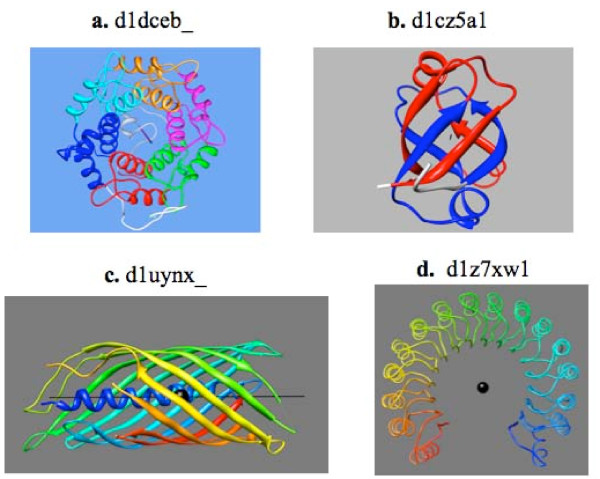
**Four more examples of symmetric domains**. (a) D1dceb_ is the Rab geranylgeranyltransferase b-chain from rat, exhibiting a 6-fold symmetric alpha/alpha toroid structure [28,29]. Each repeating unit is colored differently to show the 6 repeating units. (b) D1cz5a1 is VAT-N, the N-terminal domain of VAT protein, of the archaebacterium *Thermoplasma acidophilum*. It has a double psi beta-barrel structure with 2-fold symmetry [29]. The symmetric core of the protein is colored blue for the N-terminal half and red for the C-terminal half. (c) D1uynx_ is the outer membrane translocator domain of an autotransporter from *Neisseria meningitidis *[30]. Its structure is 12 stranded beta-barrel made of 6 up-and-down beta-hairpins. The ribbon representation is colored in rainbow colors, from blue to red for the N- to C-terminal residues. The symmetry with the highest Z-score is a 2-fold axis. But there are numerous other rotation and screw symmetries with different angles and pitches for this structure. (d) Human ribonuclease inhibitor [31], d1z7xw1. It has a leucine-rich repeat (LRR) fold. Coloring is rainbow coloring, blue to red from N- to C-terminus.

Symmetric structures can be open or closed. In a closed structure, the N- and C-termini of the molecule are physically close to each other and the symmetry is purely, or nearly purely, rotational. The amount of rotation is an integer fraction of 360°. Structures with 3- to 8-fold symmetries have been observed. These can be all alpha-helical (e.g. alpha-alpha toroid, Fig. 8a), all beta (e.g. beta-trefoil, Fig. 2b; beta-propellers, Fig. 2c) or a mixture (e.g. TIM barrel, Fig. 2d).

The 2-fold symmetric structures can be of two types. A two-fold symmetry occurs when the N-terminal and the C-terminal halves of the molecule attain a similar structure. In many such structures, the two halves fold more or less independently and form two sub-domains. The structure shown in Fig. 2a is an example of such a structure. In other cases, however, the two interact intimately over most of their length, resulting in an intertwined structure, like the double psi beta-barrel shown in Fig. 8b.

A special type is the transmembrane beta-barrels, which are made of up-and-down beta-hairpins that twist around the surface of a cylindrical barrel (Fig. 8c). These can have more than 8-fold symmetry, or more than 16 beta-strands, but all are closed structures in which the N- and C-termini come close to each other. Those with long barrels also have screw symmetries with many different rotation angles.

An open structure has a helical symmetry and the N- and C-termini of the molecule are at opposite ends of the molecule. Such a structure can be all alpha-helical (e.g. alpha-alpha superhelix, Fig. 2e), all beta-strands (e.g. beta-helix, Fig. 2f), or a mixture (e.g. leucine-rich repeats, Fig. 8d). Typically there are a large number of repeating units and the rotation angle is not an integer fraction of 360°.

**Figure 9:**
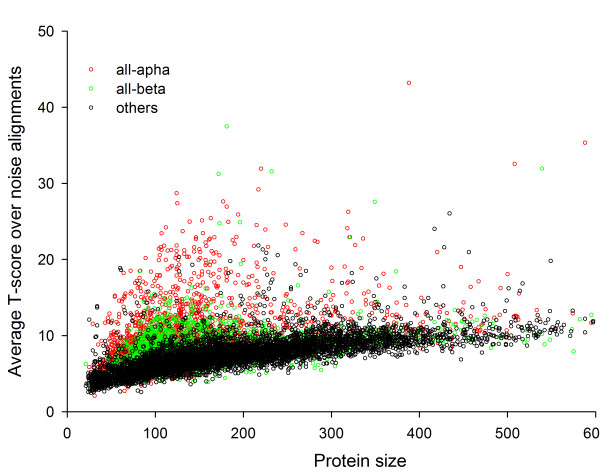
**Alignment scores of 'noise' alignments**. The alignment score averaged over all non-self 'noise' alignments for each protein is plotted against the size of the protein. All-alpha and all-beta classes are shown in red and green, respectively. These classes include alpha-helix bundles (e.g. d1f68a_) and beta-sandwiches (e.g. d1cida1), which produce high T-scores in the 100- to 200-residue range. The black points are for proteins other than all-alpha and all-beta classes.

We have seen only mono-axial symmetries in this work. But this is probably because we used the SymD algorithm to find one symmetry axis and ignored other signals that might arise from a second symmetry axis. A possible indication of the existence of a second axis for some structures is the curious high Z-scores for many 'noise' alignments seen in Fig. 9. (See Methods.) This will be the subject of a future study.

## Conclusions

We have described the principle of and the initial results obtained from the newly developed program SymD. SymD is a true symmetry detection program in contrast to many other procedures used for the same purpose but which actually simply detect structural repeats. The procedure is sensitive because (1) it allows detection of symmetry even when the structure contains symmetry-breaking insertions or deletions either within or between the repeating units and (2) it amplifies symmetric signal. The procedure yields both the sequence and structural alignments after each symmetry operation. The sequence alignment gives information on the residues that make up the repeating units. The structural alignment, or the structure transformation matrix, gives the information on the direction and position of the symmetry axis, the rotation angle, and the pitch if the symmetry is that of a helix. The procedure can detect more than one symmetry for a given molecule as described in the case of a 2-fold symmetric TIM barrel and a beta-helix structures.

A SymD run on the nearly 10,000 domains in the ASTRAL 40% domain database yielded a preliminary overall view of symmetric structures in the known domain structure world. It can be estimated that between 10% and 15% of the domains are symmetric. Many SCOP folds contain both symmetric and not-so-symmetric domains. The symmetries observed are broadly of two types, closed and open. In symmetric closed structures, the N- and C-termini of the molecule come close together and the structure has a purely rotational symmetry. Most of these have 3- to 8-fold rotational symmetries, but the transmembrane beta-barrels can have higher symmetries. In the symmetric open structures, the structure has a helical symmetry and the N- and C-termini are at the opposite ends of the molecule. Structures with a 2-fold rotational symmetry do not fit either category well; they can have either a closed (intertwined) or an open structure.

## Methods

### Alignment Scan procedure

**Figure 10:**
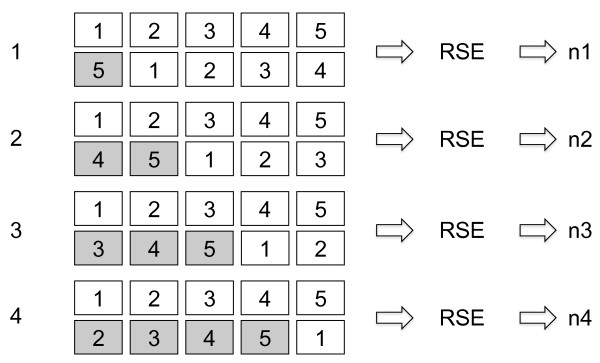
**The alignment scan procedure**. In this illustration, the protein is made of 5 residues. The first row of 5 boxes represents the original sequence. The second row of 5 boxes represents the circularly permuted sequence in which the last residue is moved to the front. This box is shaded to indicate the permutation. The initial alignment aligns residues 2, 3, 4 and 5 of the first sequence to the residues 1, 2, 3 and 4 of the second sequence, respectively. The residue 1 of the first sequence and residue 5 of the second sequence is not considered as a part of the initial alignment. This alignment is fed to the RSE routine, which refines it by structure superposition-sequence alignment cycles and produces a new, refined alignment output, indicated as *n1*. This process is repeated after circularly permuting the sequence by one more position each time. The refined alignment outputs are labeled by the number of positions of the initial permutation. This example shows that the initial alignment for the last cycle has just one pair of residues aligned. In real case, the cycle stops when the number of residue pairs in the initial alignment is less than 3.

The alignment scan procedure works as follows. First, make a duplicate of the structure of interest. Call the original structure A and the duplicate B. Then generate the *k*-th initial sequence alignment by circularly permuting the sequence of structure B by *k *residues. (See Fig. 10.) This makes residue *i *of structure B to be aligned with residue *i *+ *k *of structure A. If *i *+ *k *becomes larger than *N*, residue *i *is left unaligned in the initial alignment, where *N *is the total number of residues of the protein. Then, the RSE program is run with this initial alignment to obtain a refined structure-based sequence alignment. The procedure is repeated for all values of *k*, from 1 to *N-s*, where *s *= 3. For each value of *k*, the T-score (see below) is kept, as well as the transformation matrix for the optimal structural superposition and the refined sequence alignment.

The RSE procedure used here has been described [22]. It takes an initial sequence alignment between two structures and refines it based on structural superposition of the two structures. Briefly, the procedure consists of iterating a two-step cycle. In the first step of the cycle, the best structural superposition is obtained for the given sequence alignment by minimizing the distances between Cα atoms of aligned residue pairs using Kabsch's procedure [23,24]. In the second step, an updated sequence alignment is obtained from the superposed structures using the SE algorithm. The iteration stops when either there is no change in the sequence alignment or when a set number of iterations have been made. The final alignment reported is the one that produced the best T-score among all cycles.

The SE procedure has also been described [25]. The procedure finds a sequence alignment from a given pair of superimposed structures. It works by finding seed alignments, extending them, and then selecting a consistent set of the extended seed alignments. The procedure does not use a gap penalty and generates alignments that are generally more accurate than those that use the dynamic programming algorithm. Another virtue of SE is its speed, which enables one to run several hundred runs for a typical protein within a fraction of a second, which is required for the alignment scan procedure.

We used T-score, which is similar to the TM-score [26], as a measure of the quality of alignment. This is a weighted number of aligned residues given by the following formula:(1)

where *d*_*ij *_is the distance between the Cα atoms of two aligned residues *i *and *j*, one from structure A and the other from structure B, respectively, *d*_*o *_= 2.0 Å, and the summation is over all aligned residue pairs, except those that are *s *residues or less apart in the original sequence, where *s *= 3. This latter condition was introduced in order to discourage self-alignments.

### Finding the axis of rotation from the transformation matrix

For each refined alignment, the transformation matrix that transforms the duplicated structure B to optimally superimpose the original structure A is obtained and saved. This transformation matrix contains information on the position and orientation of the rotation axis, the rotation angle, and any translation along the rotation axis. The mathematical procedure for calculating these properties from the transformation matrix is given in the additional file 6: rotation_axis.pdf.

### The Z-score calculation

The Z-score of an alignment was calculated by comparing the T-score of the alignment against the T-score expected when the protein was not symmetrical. In order to obtain an estimate of this latter T-score, we first define the 'best' alignment for a protein as the alignment that produced the highest T-score among the *N-3 *refined alignments. Then, a refined alignment *k *is considered a 'noise' alignment if (1) the cosine of the angle between the rotation axes for the *k*-th and the 'best' alignments is less than 0.95 and (2) none of the aligned residue pairs are within *s = 3 *residues of self (i.e. *|i-j| *> *s *for all aligned residue pairs *i *and *j*). The condition (2) was included in the definition of 'noise' alignment only for the Z-score calculation.

Fig. 9 shows the average T-score (all T-scores are too many to plot) of all 'noise' alignments, defined as above, for each protein as a function of the size of the protein. It turns out that some of the all-α and all-β proteins have high 'noise' T-scores compared to others. We chose to exclude these proteins for the following curve-fitting procedure. With the remaining proteins (represented by the black points in Fig. 9), we computed the average T-score and the standard deviation of all 'noise' alignments within each 11-residue sliding window of protein sizes and then fit an exponential curve, *y = a + b(1 - exp(-cN))*, through them, where *N *is the number of residues of the protein. This procedure yielded the following size dependence:(2)(3)

where  and σ(*N*) are the average and the standard deviation of the 'noise' T-scores of proteins of size *N *residues.

The Z-score of a protein of size *N *was then obtained by(4)

### Program availability

The program SymD will be made available for download from the web site http://lmbbi.nci.nih.gov or by contacting the corresponding author.

## Authors' contributions

CK wrote the computer program and obtained most of the results. JB performed some summarizing calculations and prepared most of the figures with structural images. BL conceived of the study, supervised the research, and wrote the manuscript. All authors read and approved the final manuscript.

## Supplementary Material

Additional file 1 **all_domains**. An Excel file that gives the maximum Z-score, and the rotation angle and the translation along the rotation axis corresponding to the alignment with the maximum Z-score, for each of the 9479 ASTRAL 40 domains. Click here for file

Additional file 2 **all_folds**. An Excel file that gives the number of domains with Z-score 8 or higher, with Z-score 10 or higher, and total number of domains in each of the 1081 SCOP Folds. Click here for file

Additional file 3 **SymDGangstaData**. An Excel file that contains 8 sheets in addition to a cover sheet. Each of these 8 sheets is for a fold in Table 1 of Guerler et al. [5]. Each sheet gives FSAR (the fraction of sequentially aligned residues) from GANGSTA+, Z-score from SymD, and the ASTRAL domain name for each domain of the fold. It also gives the scatter plot between the two symmetry measures and the correlation coefficient between them. Click here for file

Additional file 4 **SymDGangsta**. A Powerpoint file that contains 8 slides, each showing the scatter plot of the SymD and GANGSTA+ symmetry measures for each fold. Click here for file

Additional file 5 **SymDGangstaTable**. An Excel file that gives a summary table of the number of domains in each fold, grouped into symmetric/non-symmetric sets by SymD/GANGSTA+. Click here for file

Additional file 6 **rotation_axis**. A PDF file that described the mathematical procedure used for obtaining the rotation angle, the translation along the rotation axis, and the position of the rotation axis from the transformation matrix for optimal structural superposition. Click here for file
