# Supplementary material for: Detecting internally symmetric protein structures
Source: BMC Bioinformatics. 2010 Jun 3;11:303. doi: 10.1186/1471-2105-11-303 (PMC2894822; doi:10.1186/1471-2105-11-303)
Supplement: Additional file 4 — SymDGangsta. A Powerpoint file that contains 8 slides, each showing the scatter plot of the SymD and GANGSTA+ symmetry measures for each fold. [file 1471-2105-11-303-S4.PPT]

## Slide 1
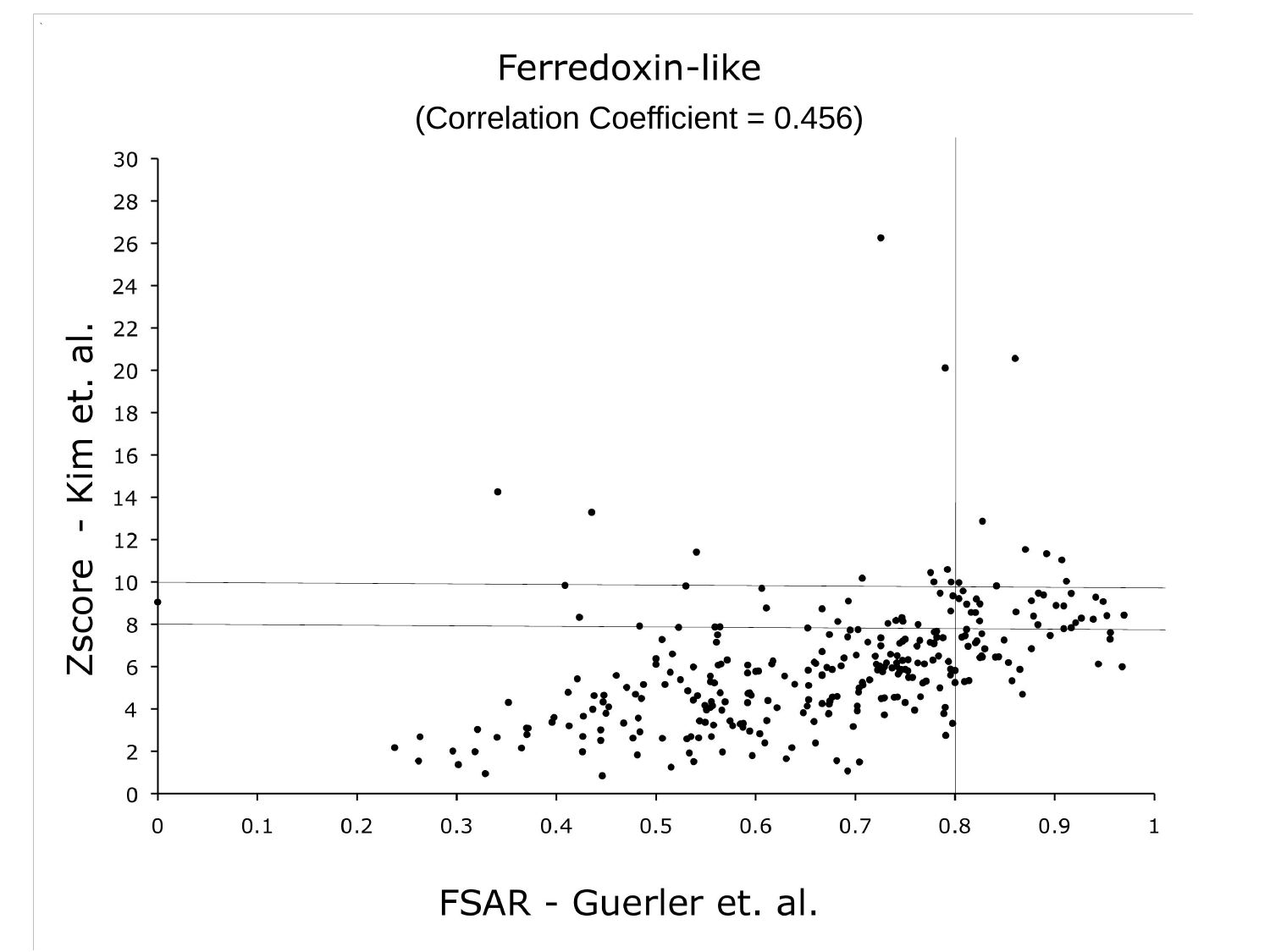

(Correlation Coefficient = 0.456)

## Slide 2
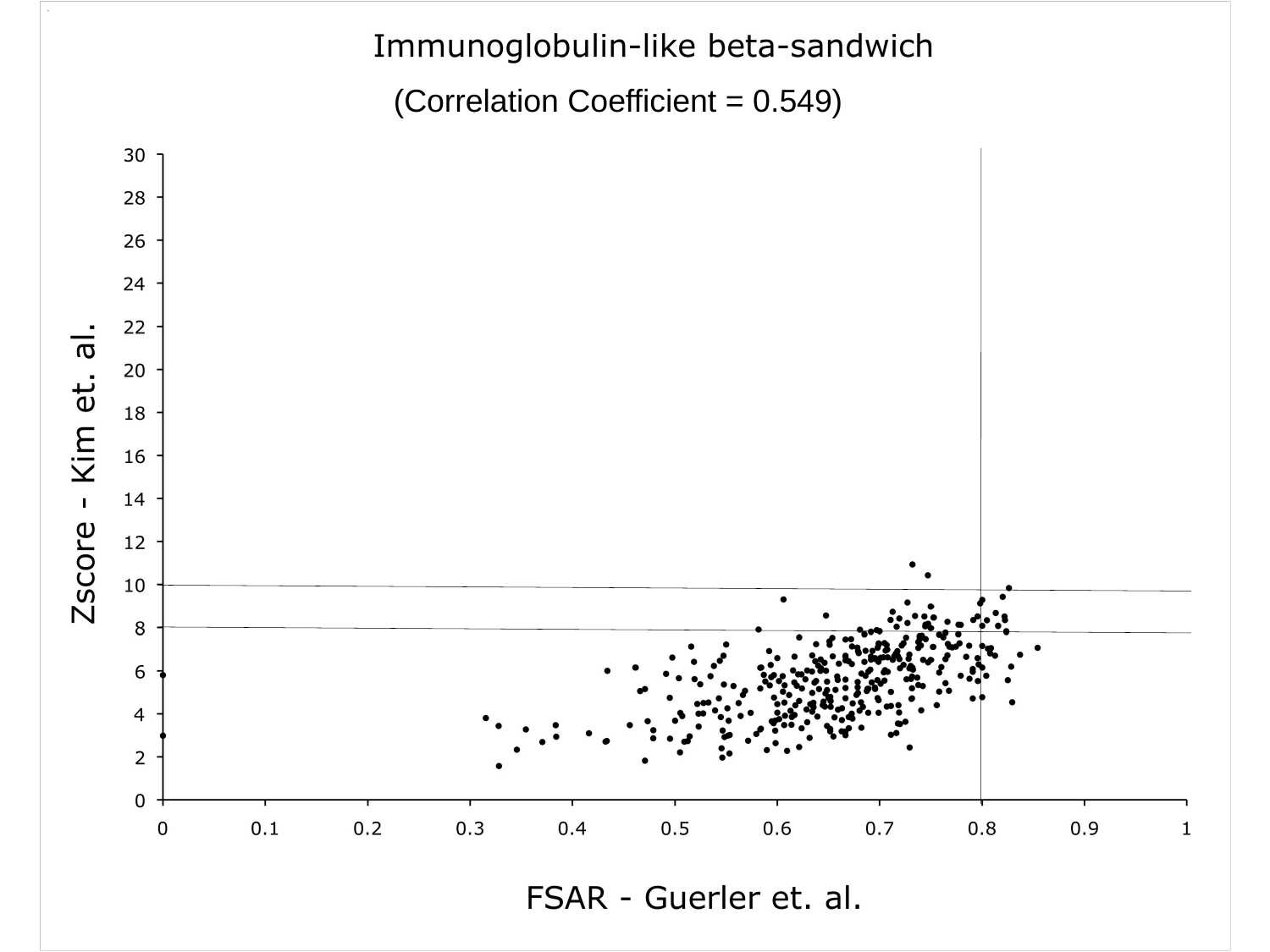

(Correlation Coefficient = 0.549)

## Slide 3
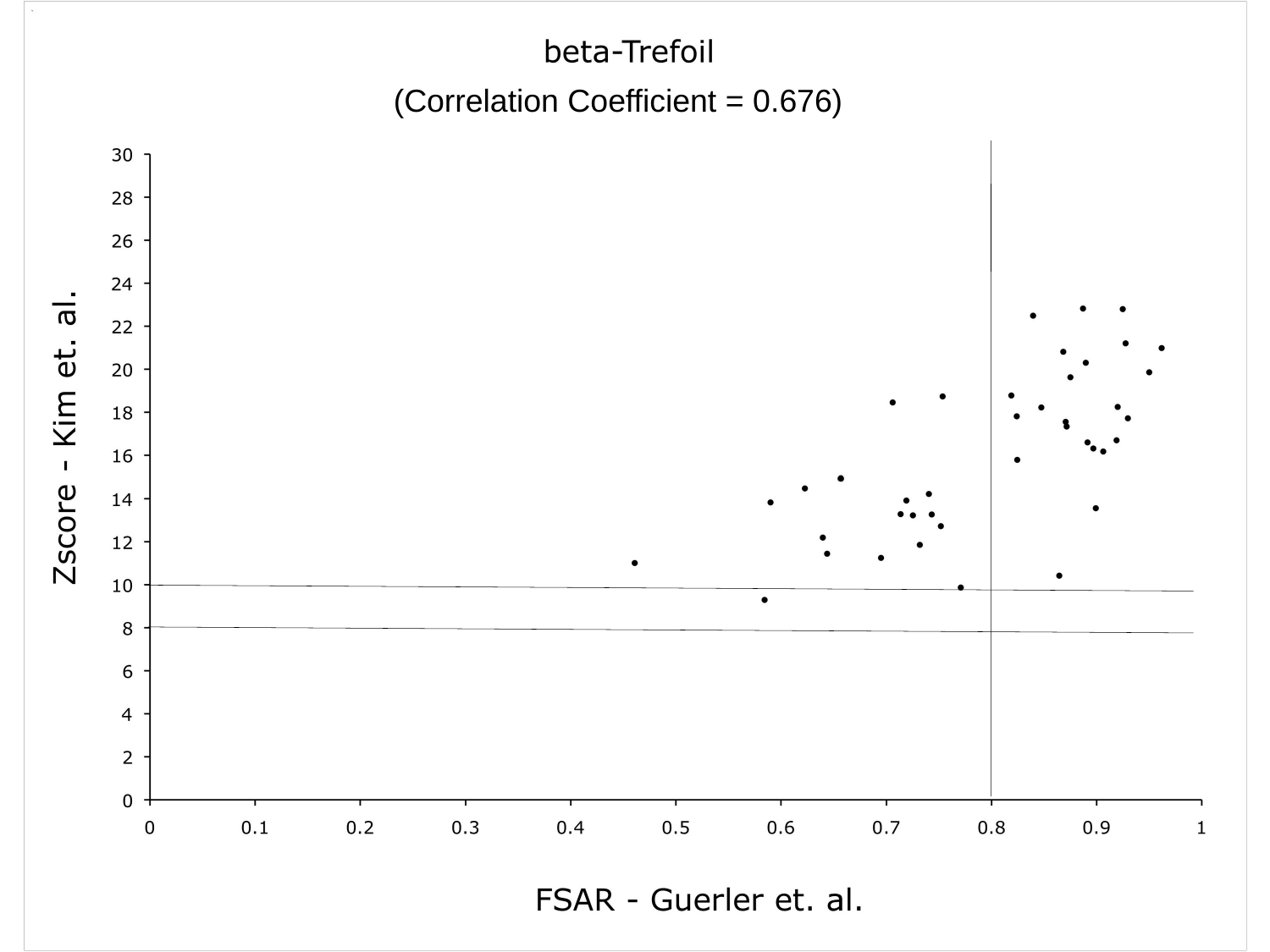

(Correlation Coefficient = 0.676)

## Slide 4
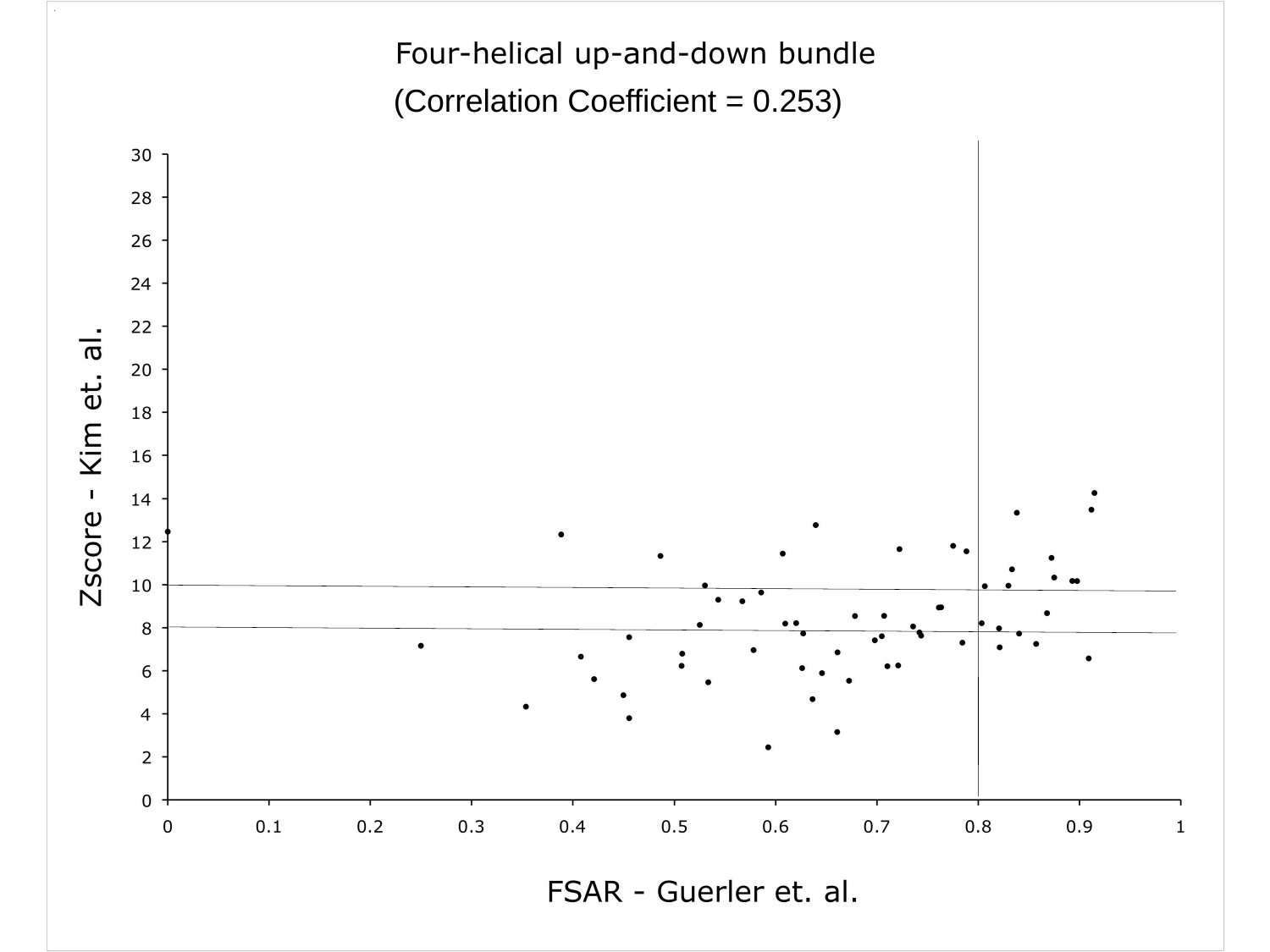

(Correlation Coefficient = 0.253)

## Slide 5
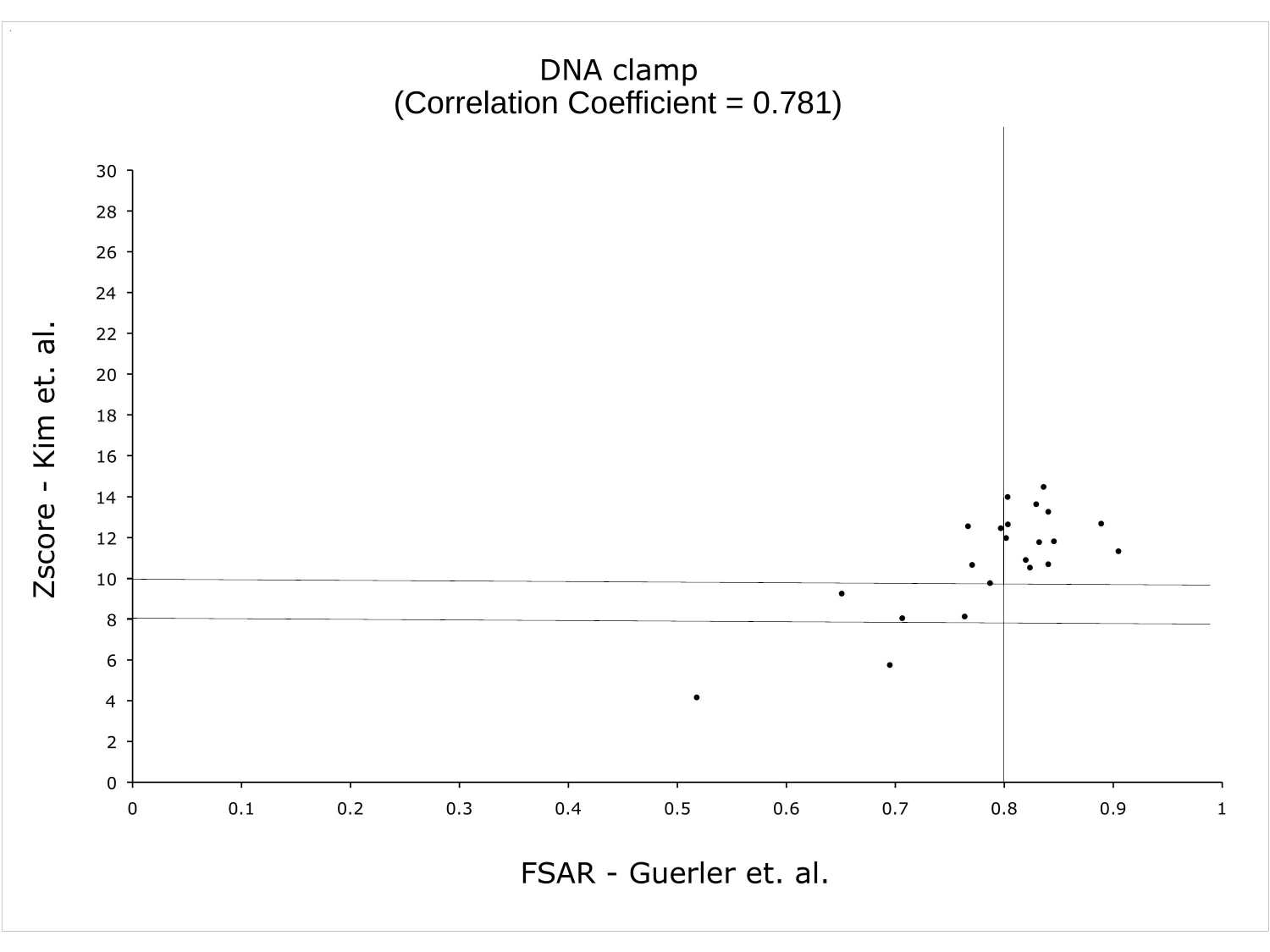

(Correlation Coefficient = 0.781)

## Slide 6
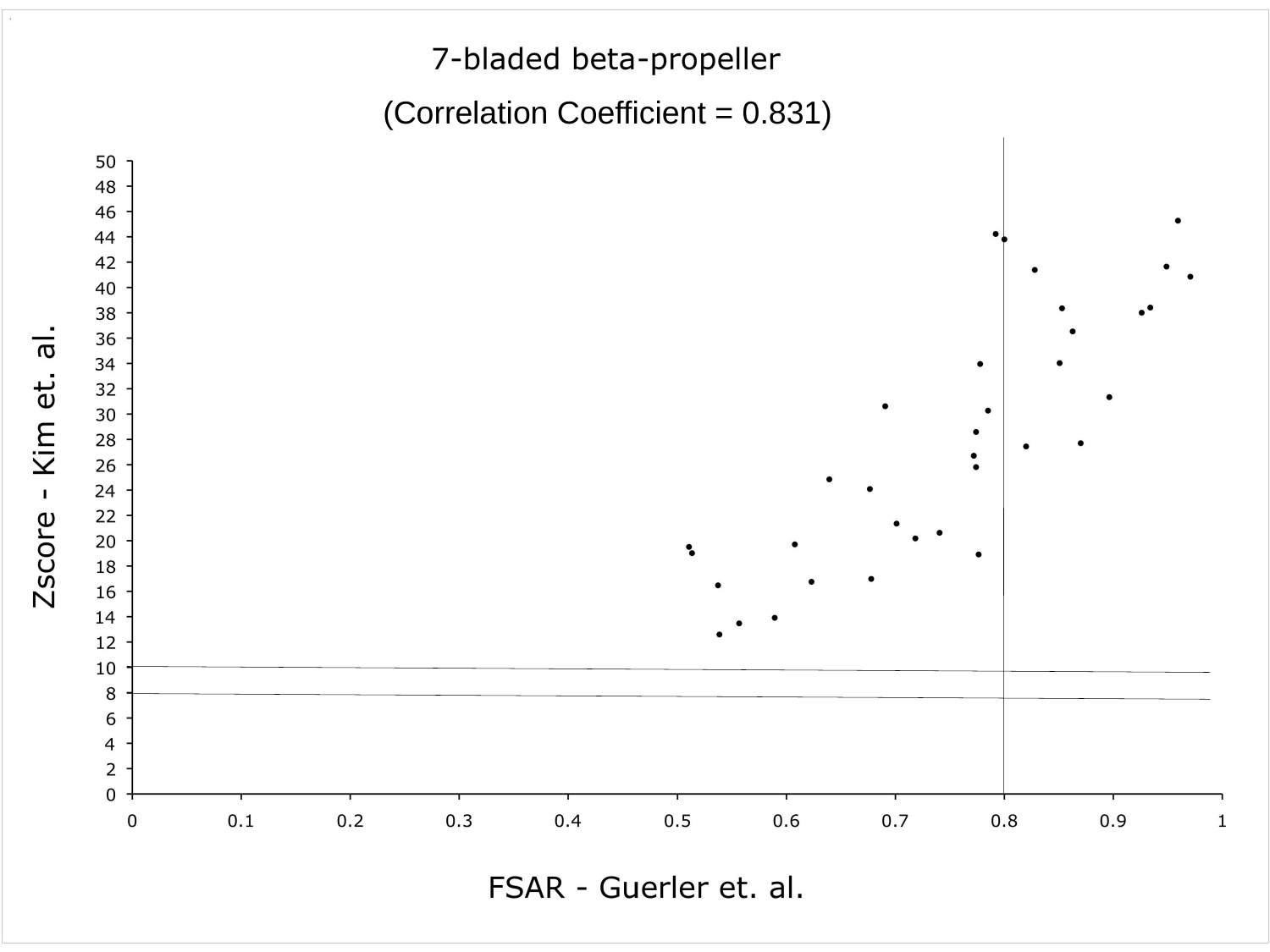

(Correlation Coefficient = 0.831)

## Slide 7
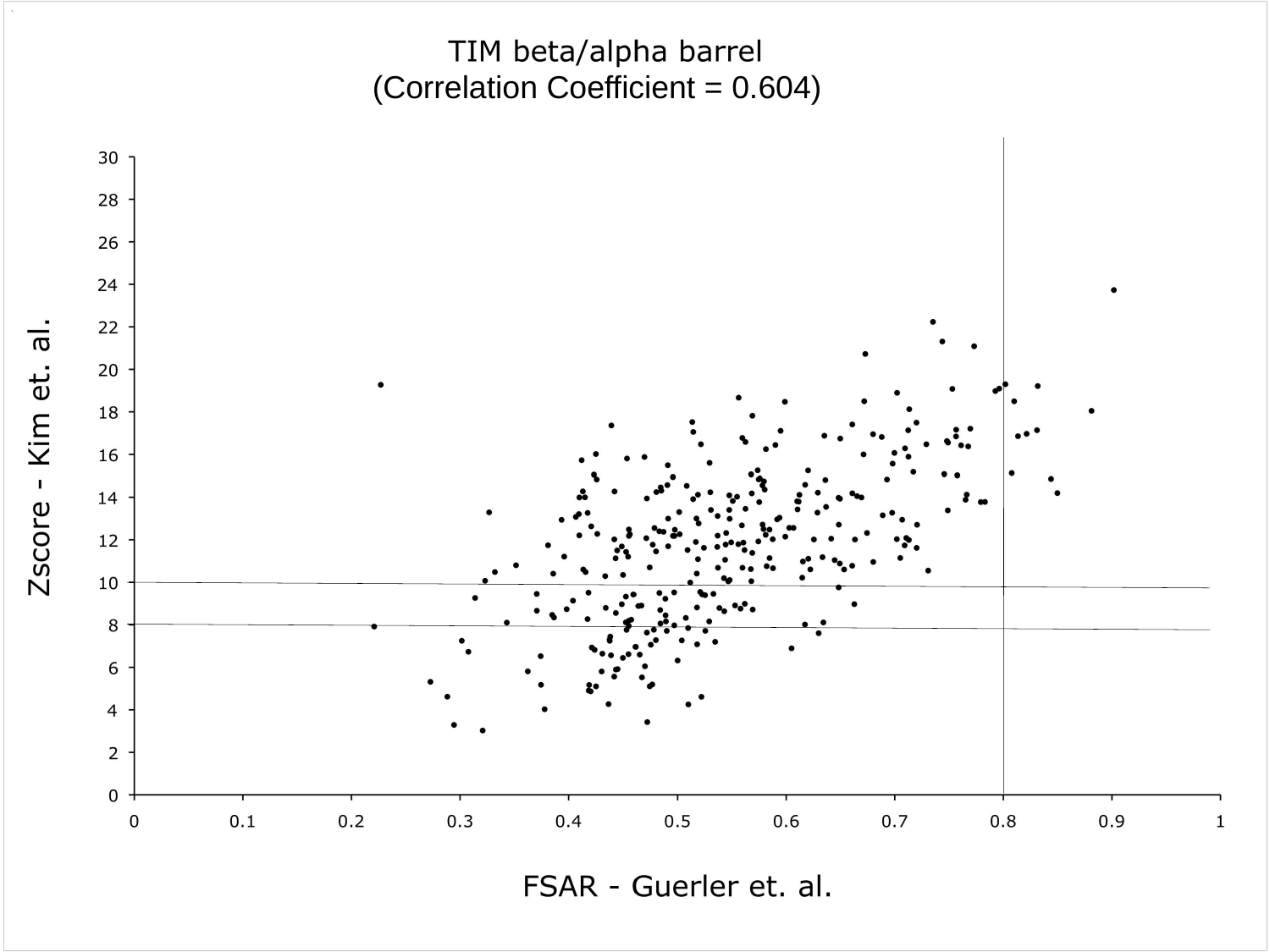

(Correlation Coefficient = 0.604)

## Slide 8
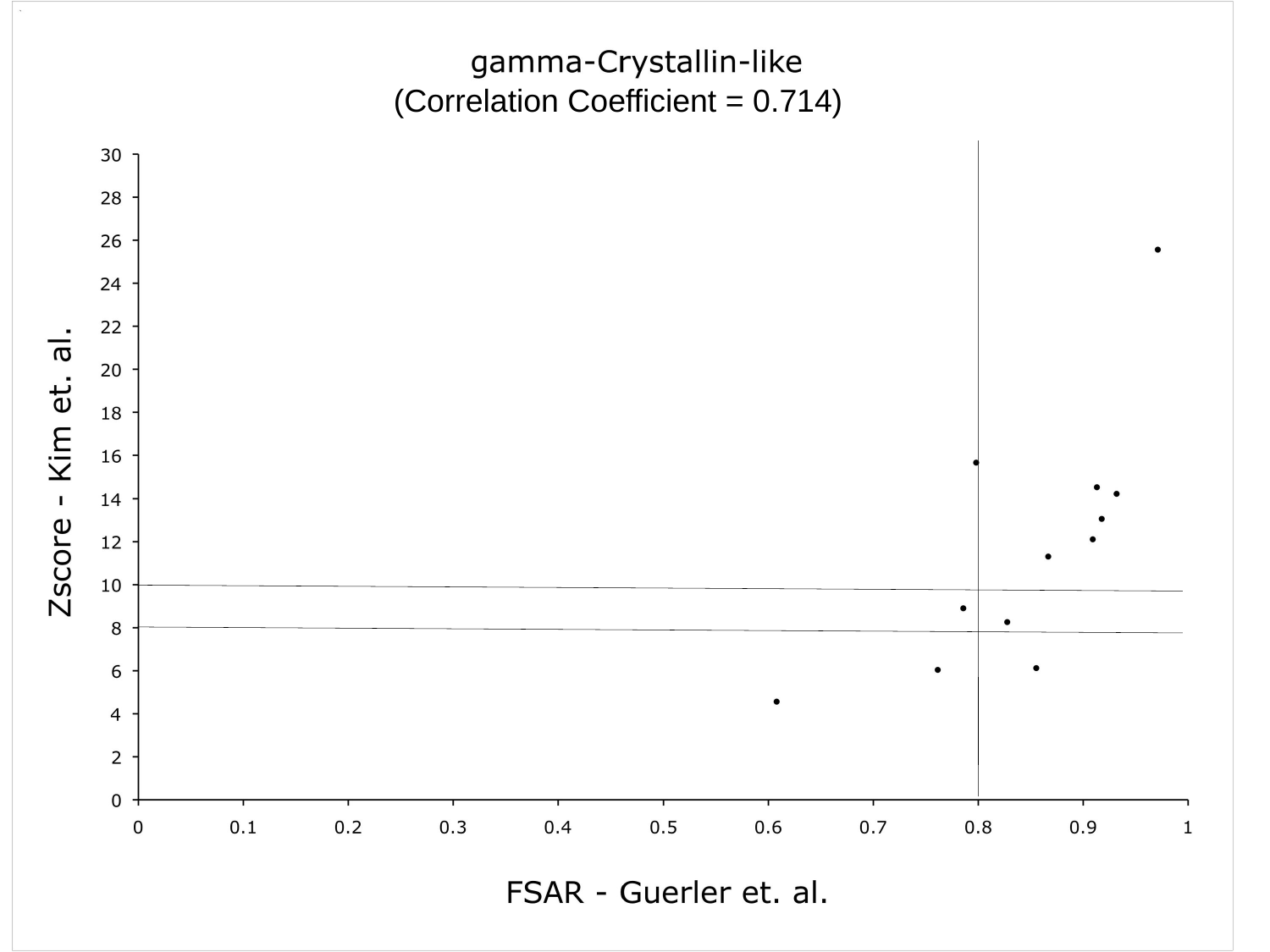

(Correlation Coefficient = 0.714)
