# Supplementary material for: Detecting internally symmetric protein structures
Source: BMC Bioinformatics. 2010 Jun 3;11:303. doi: 10.1186/1471-2105-11-303 (PMC2894822; doi:10.1186/1471-2105-11-303)
Supplement: Additional file 6 — rotation_axis. A PDF file that described the mathematical procedure used for obtaining the rotation angle, the translation along the rotation axis, and the position of the rotation axis from the transformation matrix for optimal structural superposition. [file 1471-2105-11-303-S6.PDF]

Appendix: Finding the axis of rotation from the transformation operator

Let α be a transformation operator. It consists of a translation vector ***t*** and a rotation matrix ***R***, i.e., $\alpha = (\boldsymbol{t}, \boldsymbol{R})$, such that a position ***r*** is transformed to a new position ***r'*** by

$$\boldsymbol{r}' = \alpha \boldsymbol{r} = \boldsymbol{t} + \boldsymbol{R}\boldsymbol{r}. \qquad (1)$$

Such a transformation operator is a primary output of the structure-structure alignment programs.

Given such a transformation matrix, the task is to find the position and orientation of the rotation axis, the rotation angle, and any translation component along the rotation axis. (When there is a non-zero translation along the rotation axis, the combined operation of a rotation followed by a translation along the rotation axis is called a screw operation. A helix results when such an operation is repeated.)

1. The rotation angle and the direction of the rotation axis

The matrix ***R*** can be written in terms of the rotation angle θ and the unit vector ***u*** along the rotation axis as follows (http://en.wikipedia.org/wiki/Rotation_matrix):

$$R = \begin{bmatrix} u_1^2 + (1-u_1^2)c & u_1u_2(1-c) - u_3 s & u_1u_3(1-c) + u_2 s \\ u_1u_2(1-c) + u_3 s & u_2^2 + (1-u_2^2)c & u_2u_3(1-c) - u_1 s \\ u_1u_3(1-c) - u_2 s & u_2u_3(1-c) + u_1 s & u_3^2 + (1-u_3^2)c \end{bmatrix}, \qquad (2)$$

where $c = \cos\theta$, $s = \sin\theta$, and u_1, u_2, u_3 are the x, y, z-components of the vector ***u***.

From the sum of the diagonal elements of this matrix, one obtains

$$\cos\theta = (R_{11} + R_{22} + R_{33} - 1)/2. \qquad (3)$$

This gives the rotation angle except for the sign.

When the rotation angle is not zero or very small, the diagonal elements of the rotation matrix can be used to obtain

$$|u_i| = \sqrt{\frac{R_{ii} - c}{1 - c}}. \qquad (4)$$

However, this formula can give inaccurate values when the angle is small ($c \approx 1$). In practice, it is better to calculate them by

$$v_i = \sqrt{R_{ii} - c} \qquad (4a)$$

and

$$|u_i| = v_i / \sqrt{v_1^2 + v_2^2 + v_3^2} \qquad (4b)$$

The signs of u_i and of the angle θ can be obtained from the difference of the off-diagonal elements:

$$R_{32} - R_{23} = 2u_1 s$$
$$R_{13} - R_{31} = 2u_2 s \qquad (5)$$
$$R_{21} - R_{12} = 2u_3 s \; .$$

If we choose the positive sign for the angle, s is positive and the signs of u_i are the same as those of the corresponding off-diagonal element differences. If we choose the negative angle, then the sign of u_i changes and the vector $\boldsymbol{u}$ points to the opposite direction. Since rotating by a positive angle around an axis is the same as rotating by a negative angle around the flipped axis, the two choices result in the same solution and we may chose either one of the two procedures. Therefore, we will arbitrarily chose the sign of the angle θ as positive and then choose the direction of $\boldsymbol{u}$ according to (5).

Equations (5) determine the sign of all u_i. However, when angle θ is 180° or close to it (or 0°, but we will handle zero-angle case separately), s is zero or nearly zero, which means that the off-diagonal term differences are zero or nearly zero, i.e. the matrix $\boldsymbol{R}$ is exactly or nearly symmetric. In such cases, determining the sign of $\boldsymbol{u}$ by (5) may become unstable. Instead, using the sum of the off-diagonal elements will produce more stable answers:

$$R_{12} + R_{21} = 2u_1u_2(1-c)$$
$$R_{13} + R_{31} = 2u_1u_3(1-c) \qquad (6)$$
$$R_{23} + R_{32} = 2u_2u_3(1-c) \; .$$

Thus, the recommended procedure is to choose the sign of only one, the largest, component of $\boldsymbol{u}$ (one with the largest magnitude) using one (the one with the largest difference) of the equations (5) and then use equations (6) to determine the signs of the remaining two terms of $\boldsymbol{u}$. This procedure can be used generally even when the angle is not nearly 180°. When the angle is exactly 180°, the same procedure can be used, even though the differences in the equations (5) are all zero, because sign of the angle becomes irrelevant in this case (180° = -180°) and the sign of one component of $\boldsymbol{u}$ can be chosen arbitrarily.

When the rotation angle is zero or very small, the rotation matrix $\boldsymbol{R}$ is exactly or nearly identity, c is 1 or nearly 1, and eqs. (4), (4a) and (4b) will give unstable values for the magnitudes of u_i. In this case, however, the transformation is purely or nearly purely translational and the rotation axis is ill-defined. Therefore, we arbitrarily choose $\boldsymbol{u}$ to be parallel to $\boldsymbol{t}$ and choose the sense of $\boldsymbol{u}$ according to the largest component of the equations (5).

2. The position of the rotation axis when the angle is not too small

The translation vector $\boldsymbol{t}$ of α can be separated into two components, one parallel and the other perpendicular to the rotation axis:

$$t = t_{\parallel} + t_{\perp} \, , \qquad (7a)$$

with

$$\boldsymbol{t}_{\parallel} \bullet \boldsymbol{t}_{\perp} = 0 \qquad (7b)$$

and

$$\boldsymbol{R t}_{\|} = \boldsymbol{t}_{\|} . \qquad (7c)$$

When **u** is known,

$$t_{//} = (t \bullet u)u \qquad (8a)$$

and

$$t_{\perp} = t - t_{//} . \qquad (8b)$$

The $\boldsymbol{t}_{\|}$ vector gives the screw component and is invariant of the sign of $\boldsymbol{u}$.

The $t_{\perp}$ vector contains information on the position of the rotation axis, but not directly. The position of the rotation axis is given by **s**, which is the vector from the origin of the coordinate system to the rotation axis and perpendicular to the axis. The relation between $t_{\perp}$ and **s** is illustrated in Figure 1.

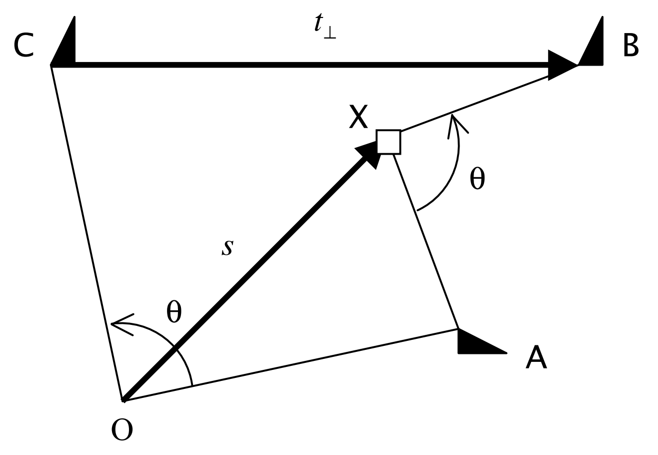

Figure 1. Relation between the translation component of the transformation operator and the position of the rotation axis. The view is down the rotation axis, which is indicated by an open square at position X. The origin of the coordinate system is at the position marked O. The vector **s** from O to X gives the location of the rotation axis. Object A is moved and rotated to object B by the rotation at X by an angle θ. The same object B can be obtained from A by first rotating A around the axis at the origin by the same angle θ, followed by a translation $t_{\perp}$.

Let B and C be the objects obtained after object A is rotated around the rotation axes located at positions X and O, respectively, O being the origin of the coordinate system. A point in object A is transformed by the operator α to a point in object B as

$$r_B = \alpha r_A - t_{\|} = t + R r_A - t_{\|} = t_{\perp} + r_C . \qquad (9)$$

But $\boldsymbol{r_B}$ can also be obtained from $\boldsymbol{r_A}$ by first moving the origin of the coordinate system to X and then rotating it by $\boldsymbol{R}$:

$$r_B = R(r_A - s) + s = Rr_A - Rs + s\,. \qquad (10)$$

Comparing eqs. (9) and (10), one obtains

$$t_\perp = s - Rs\,. \qquad (11)$$

(**1-R**) is a projection operator, which projects all vectors to a plane perpendicular to the rotation axis. Such an operator has no inverse. However, ***s*** can be obtained from $t_\perp$ from the geometry shown in Figure 2.

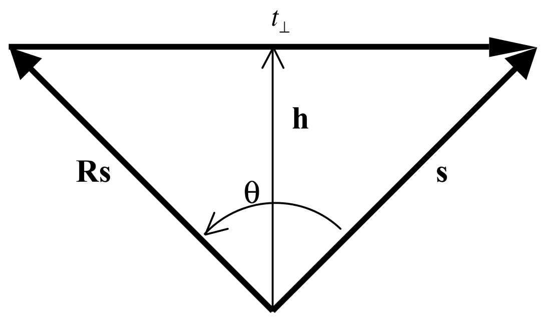

Figure 2. Determining **s** from $t_\perp$. The triangle is an isoceles triangle with base length $t_\perp$ and apex angle *θ*. The view is down the rotation axis vector ***u***.

Let **h** = (**s** + **Rs**)/2. From Figure 2,

$$h = \frac{(u \times t_\perp)}{|t_\perp|}\frac{|t_\perp|/2}{\tan(\theta/2)} = \frac{(u \times t_\perp)}{2\tan(\theta/2)}\,. \qquad (12)$$

Then **s** is given by

$$s = h + t_\perp/2\,. \qquad (13)$$

When the angle is zero or small, *h* cannot be determined from (12).

Equations (12) and (13) are, nevertheless, useful when the angle is not too small and particularly in the context of crystallographic symmetry. The angle β between **s** and $t_\perp$ vectors is given by

$$\beta = 90° - \theta/2. \qquad (14)$$

Examples:

2-fold rotation:

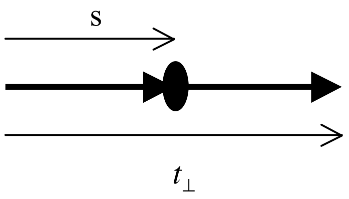

$\theta = 180°$

$\beta = 0, \ |h| = 0, \ |s| = |t_\perp| / 2$

3-fold rotation:

$\theta = 120°$

$\beta = 30^\circ, \ |h| = \frac{1}{2\sqrt{3}}|t_\perp|, \ |s| = \sqrt{\left(\frac{1}{2}\right)^2 + \left(\frac{1}{2\sqrt{3}}\right)^2}\,|t_\perp|$

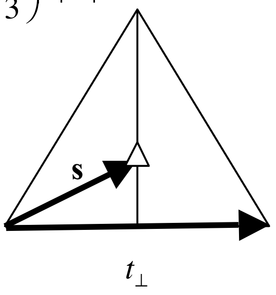

4-fold rotation:

$\theta = 90°$

$\beta = 45^\circ, \ |h| = \frac{1}{2}|t_\perp|, \ |s| = \frac{\sqrt{2}}{2}|t_\perp|$

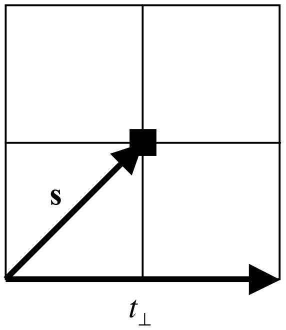

3. The position of the rotation axis when the angle is small

Above procedure for determining the position of the rotation axis cannot be used when the angle is small. Generally, the position determined by (13) will carry large error when the angle is small. It can be shown that

$$\frac{\partial |s|}{\partial |t_\perp|} = \frac{1}{2\sin(\theta/2)}, \quad (15)$$

which becomes infinite as θ approaches zero. Therefore, eq. (13) should be used only when the angle is sufficiently large.

When the rotation angle is zero or small, the operation is pure, or nearly pure, translation and the rotation axis is ill-defined. The exact placement of the axis then becomes unimportant as long as it is near the object being transformed. Therefore, we will simply place the rotation axis at the center of the fixed object when the rotation angle is smaller than a cutoff value. Some trials with the beta-helices suggest that the cutoff value should be about 5°.

Often the transformation operator α is given in the following form:

$$\boldsymbol{r'} = \alpha \boldsymbol{r} = \boldsymbol{t_1} + \boldsymbol{R}(\boldsymbol{r} - \boldsymbol{t_2}), \quad (1a)$$

where $\boldsymbol{t_1}$ and $\boldsymbol{t_2}$ are the centroids of the fixed and moving objects, respectively. In such a case, we will put the rotation axis is at $\boldsymbol{t_1}$.

References

1. http://en.wikipedia.org/wiki/Rotation_matrix

2. For many examples of new symmetry axes generated by the combination of a rotation axis at the origin and a translation, see *International Tables for Crystallography, Volume A: Space group symmetry*, http://it.iucr.org/A/.
